# Hospital heterogeneity: what drives the quality of health care

**DOI:** 10.1007/s10198-017-0891-9

**Published:** 2017-04-24

**Authors:** Manhal Ali, Reza Salehnejad, Mohaimen Mansur

**Affiliations:** University of Manchester, Manchester, UK

**Keywords:** Health care quality, Stroke, NHS, Machine learning, Regression trees, Panel data, Prediction, Mixed effects model, C23, C40, C52, C53, D23, I10, ILL, L22

## Abstract

A major feature of health care systems is substantial variation in health care quality across hospitals. The quality of stroke care widely varies across NHS hospitals. We investigate factors that may explain variations in health care quality using measures of quality of stroke care. We combine NHS trust data from the National Sentinel Stroke Audit with other data sets from the Office for National Statistics, NHS and census data to capture hospitals’ human and physical assets and organisational characteristics. We employ a class of non-parametric methods to explore the complex structure of the data and a set of correlated random effects models to identify key determinants of the quality of stroke care. The organisational quality of the process of stroke care appears as a fundamental driver of clinical quality of stroke care. There are rich complementarities amongst drivers of quality of stroke care. The findings strengthen previous research on managerial and organisational determinants of health care quality.

## Introduction

**Fig. 1:**
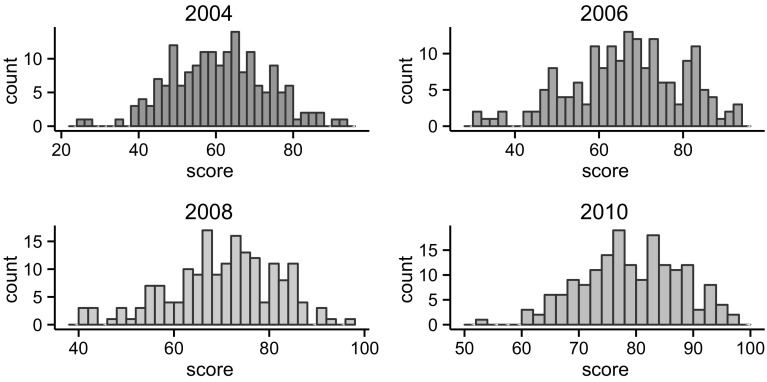
Histogram of the total process score

A central observation of the health care systems is the existence of substantial heterogeneity in the quality of health care services across hospitals. The quality of health care measured along dimensions such as mortality or the process of care for medical conditions such as cancer, stroke and pneumonia varies widely across hospitals [3, 42]. Figure 1 captures the distribution of the quality of the process of care for stroke across NHS hospitals or trusts using the data from the Royal College of Physicians for the years 2004–2010.

It is evident that the quality of the process of stroke care varies across hospitals. The figure also reveals a notable positive shift in the distribution of the quality of stroke care over the years. Notwithstanding this, variations remain, with some hospitals still performing above the median quality whereas others are performing below the median quality. Mooney [55] and Rudd et al. [69] reveal wide variations in the quality of stroke care across the regions in the UK and England. Mohammed et al. [54] also document similar variations in the case of NHS hospitals. And Ayanian and Weissman [3] and Kupersmith [42] provide a systematic review on the differences in health care quality across teaching and non-teaching hospitals.

Hospitals are major providers of health care services and account for a major expenditure of the overall health care budget. Any effort at reducing the heterogeneity and enhancing the quality of care across hospitals can bring substantial benefits to society. Such efforts demand understanding why some hospitals perform better than other hospitals, providing a higher quality of care.

The literature on the drivers of hospital quality is vast, offering a rich list of explanations for the observed heterogeneity in the quality of care. We borrow a theoretical framework from the economic literature on productivity [75] to shape our analysis. Using the framework, we distinguish between internal and environmental drivers of the quality of health care. Internal drivers refer to factors that are under the control of the hospital’s management team. The literature studying internal drivers has paid most attention to hospital size, physical assets, human capital, financial constraints, IT investment, R&D and organisation of decisions. External drivers refer to competition in the output market, regulatory environment and general socio-economic conditions.

Starting with the empirical literature on the internal drivers of hospital performance, a series of papers studies impacts of hospital size, physical assets, human capital and information technology on the quality of health care. Raerty et al. [64] and Needleman et al. [57] study the role of human capital and find a positive relationship between nurse staffing and health care quality in NHS and US hospitals. Athey and Stern [2] and Menachemi et al. [51] provide evidence on a positive relationship between health information technology and outcomes. Information technology reduces communication costs, facilitates coordination across diverse and complex activities and assists information processing and decision making. All these can reduce errors and enhance quality. Gaynor et al. [30], O’Brien et al. [58] and Hentschker and Mennicken [34] document a positive relationship between volume and outcome and process measures of quality of care. The association is likely due to the ability of larger hospitals to capitalise on the economies of scale and benefit from specialisation that can enhance the quality of care.

An emerging finding in the literature is that the above factors do not fully account for the observed heterogeneity. Variations in care measured in mortality for disease conditions or the process of care measures persist even after controlling for case mix, human capital (medical and non-medical staff), information technology, hospital characteristics such as bed size, teaching status or foundational trust status, cost–contributing factors, post-operative complications and statistical noise [5, 10, 11, 31, 35, 47, 68].

The finding has led researchers to turn attention towards management practices and organisational factors. Bloom et al. [8] collect survey data on management styles and practices from 2000 hospitals across the UK, USA, Canada, France, Germany, Sweden, Italy, Brazil and India and join the survey data with hospital performance data. The researchers find that the quality of management practices and hospital performance are highly correlated, suggesting a possible role of management practices in driving hospital outcomes. Bray et al. [12] study the possible impact of the organisation of the provision of care on the quality of stroke care, using the data on English NHS trusts for the year 2010. The authors employ a composite measure of the quality of the organisation of stroke services by taking into account staffing (e.g., training level), facilities (e.g., provision of continuous physiological monitoring) and service level (e.g., access to round-the-clock emergency imaging and thrombolysis). Adjusting for patient characteristics, the researchers find that hospitals with higher organisational scores are more likely to achieve higher clinical quality measured by stroke process of care measures. Flood [25], West [77], McConnell et al. [48, 49] and Ghaferi et al. [31] also highlight the importance of managerial and organisational practices as an underlying cause of the variations in the quality of care across hospitals. The increasing evidence on the critical role of managerial and organisational factors points to the view that it is the organisation of resources and management routines that eventually link hospital inputs to outputs. There is no production function independent of the organisation of the firm or, in our case, hospital [46]. As the organisational characteristics of a hospital change, so does the hospital’s production function.

Another substantial body of literature examines the role of external factors in shaping hospital performance. A key result in this literature is that competition, provided prices are regulated, has a positive impact on health care quality. NHS hospitals in more competitive regions provide higher health care quality compared to hospitals located in regions where there is less competition [61]. In the same line, Bijlsma et al. [4] study the impact of competition introduced in The Netherlands from 2004 to 2008 on a wide range of quality measures. The results indicate that hospitals in areas with stronger competition show higher improvement in several process measures of quality but the impact of competition on outcome measures appears negligible. Other researchers have looked, among other factors, at the impact of government policies on mergers [29], public reporting of performance [44] and provision of information [38].

This study seeks to contribute to the literature in health economics that argues for the impact of management practices and organisational factors in driving hospital performance and the quality of clinical care, where by organisational factors we also mean organisation of resources [18, 75]. Building on the literature, our general hypothesis is that hospital performance is not only dependent on the overall availability of resources but, more critically, depends also on the organisation and management of the resources and the fit between the resources and management practices [18, 28]. Variations in the organisation and management of resources partly account for the observed heterogeneity in the quality of care across hospitals [19]. The significance of such factors is likely to be higher for some of the services a hospital provides, such as provision of care for stroke patients [12]. Stroke is a medical emergency that requires immediate medical intervention as the benefit to the patient is time dependent. To achieve this, a high level of coordination is required between various steps of care from the onset of stroke to rehabilitation. And this is only feasible if relevant complementary resources are in place and well organised and stroke specialists and trained staff collaborate in a multidisciplinary and timely manner. As an attempt to further investigate the general hypothesis, we focus on the provision of stroke care. Our specific hypothesis, thus, is that variations in the management and organisational quality of the process of hospital services such as stroke care partly drive variations in the clinical quality of stroke care.

Our emphasis on the organisation of resources and management practices is by no means new. A substantial body of literature, as mentioned above, has already documented evidence on the possible impact of organisational and management factors in driving hospital performance. The literature, however, has so far mainly sought to identify factors that drive the quality of care and has paid little systematic attention to possible interactions among the factors driving hospital performance. This is partly because the theory is silent on complex interactions that may exist among key variables driving performance and partly because common econometric techniques are not equipped to reveal interactive structures based on the data alone. The theory, though, emphasises the importance of complementarity among assets and skills that drive productivity and, in our case, health care quality. In other words, it points to the importance of critical interactions among factors driving quality, not simply the mere presence or absence of the factors or the level of the factors [14]. We aim to take a first step towards systematically exploring interactive structures (complementarities) among the variables that drive hospital quality in a data-driven manner. With this in mind, our research hypothesis runs as:

*Research hypothesis*: Variations in the organisation and management of resources involved in the process of hospital services such as stroke care partly drive variations in the clinical quality of the services (stroke care). Moreover, it is the *joint* presence of complementary resources and management practices that critically affects the quality of the clinical process of the services (stroke care).

To assess this hypothesis, we put together a wide panel data set on a rich list of candidate variables that could possibly drive hospital quality in stroke care for which the organisation of resources and management of the process of care matter critically. The panel spans from year 2004–2010. To our knowledge the only significant policy change that took place during this period is the introduction of Department of Health’s National Stroke Strategy implemented in England in December 2007. We appropriately account for this change in our analysis in order to capture the potential impact this policy may have on the quality of stroke care in English hospitals. As our measure of quality, we employ an index that captures the quality of the process of stroke care as opposed to an outcome measure. Process of care measures illuminate the complicated process of delivering health care and describe the specific actions associated with health care delivery. It describes the care that patients actually receive and assesses the extent to which hospitals perform health care processes to achieve the desired aims. An important advantage of process measures over outcomes is that it does not suffer from the case-mix adjustment bias [12, 22]. Outcome measures such as mortality, in contrast, may be confounded by variations in demographics or case mix and may be susceptible to coding or measurement errors. However, the validity of process measures depends on whether they are a useful proxy of subsequent outcomes and that the interventions being measured are related to the expected effects [76].

We combine our measure of the clinical quality of stroke care with a measure of the organisational quality of the process of care and other data drawn from several sources. We will employ a new class of unbiased panel regression tree estimator from the machine learning literature to study the data. A reason behind the choice of the method is the intuitive interpretability of the results. The non-parametric method can reveal potential interactions among the variables, which could offer valuable information about the processes that drive variations in quality of stroke care across NHS hospitals. We use an out of sample prediction error estimator to calculate the predictive accuracy of the models.

Our results point to the significant role of organisational features of the process of stroke care in driving the clinical quality of the process of stroke care. Including a composite measure of organisational design for the provision of stroke care improves the predictive accuracy of the models. The predictive results are robust to the inclusion of year as an additional variable to capture trends in the data. Using year as an additional predictor, our measure of organisational design for the provision of stroke care still has a significant predictive role in that it reduces both the in-sample and out-of-sample prediction error rate. Furthermore, the results of the unbiased panel regression tree estimator are consistent with our benchmark linear mixed effects model. The coefficients in the linear mixed effects model are consistent with the variables entering in the tree models. Specifically, the coefficient of the organisation score is substantial and statistically significant. The results are robust to the inclusion of fixed hospital and year effects. The key result also remains unchanged when we rescale the clinical and organisational measures of quality by taking out the within-hospital means of the variables to eliminate potential hospital-specific unobserved time-invariant factors.

Our findings are consistent with the theories from the organisational economics and productivity literature when extended to the health care setting. The results are particularly in line with the findings of Bloom and Van Reenen [9], Bloom et al. [6] and Bloom et al. [7] who find that capital intensity or technology cannot alone fully account for large differences in total factor productivity (TFP) across firms. Management practices and organisational design are most fundamental in driving hospital performance and quality. High organisational quality enhances performance, productivity and quality. At the heart of any policy effort to eliminate the observed heterogeneity in the quality of care across hospital there should be a well-founded effort to reduce heterogeneity in organisational quality and management practices.

The rest of the article is organised as follows. "Data and descriptive statistics" introduces the data. "Panel regression trees" outlines the predictive approach and describes a class of non-parametric regression methods for the study of panel data. "Empirical analysis" presents our basic empirical results by assessing the predictive accuracy of the hypotheses. "Robustness analysis" carries out robustness checks. "Conclusion" concludes the analysis.

## Data and descriptive statistics

The study has gathered hospital and regional level panel data from several administrative sources. Our two key variables include composite measures of the clinical and organisational quality of the process of stroke care. We obtain the data on these measures from the Royal College of Physician’s National Sentinel Stroke Audit from 2004 to 2010 where the data have been collected at the hospital level. The Royal College of Physicians carried out rounds of the National Sentinel Stroke Audit (NSSA) in every 2-year cycle from 1998 to 2010. The NSSA monitored the progress of stroke care in England, Wales and Northern Ireland. Between 1998 and 2010, the NSSA achieved 100% voluntary participation of hospitals, collecting data on more than 60,000 patients from England, Wales and Northern Ireland. The focus of the audit was centred on two components: clinical and organisational. The former measured the clinical quality of the process of stroke care whereas the latter measured the organisational quality of stroke care. NSSA was eventually replaced by Sentinel Stroke National Audit Programme (SSNAP). As a result of the replacement, some of the questions used to construct the organisational and clinical components of the NSSA data underwent changes, making it difficult to combine the data from the two audits. For consistency, we have restricted the sample to the NSSA data that are in public domain and left the SSNAP data for a separate research. The audit data have been used for research studies, for instance, in Bray et al. [12] and McNaughton et al. [50].

### Clinical process of care

In assessing the quality of the process of care for stroke, the NSSA collected data on the practice of a set of clinical standards relating to patients hospitalised with a primary diagnosis of stroke with the ICD10 codes of I61 (intracerebral haemorrhage), I63 (cerebral infarction) or I64 (stroke, not specified as haemorrhage or infarction). Data were collected on 8697, 13,625, 11,369 and 11,353 patients respectively for the years 2004, 2006, 2008 and 2010 across the NHS trusts or hospitals in England, Wales and Northern Ireland. The clinical standards reflect scientifically based practices that are set by the National Institute for Health and Clinical Excellence (NICE). The clinical standards are organised into six domains of the process of stroke care, including initial patient assessment, multidisciplinary assessment, screening and functional assessment, care planning, communication with patients and carers, and acute care. Table 4 lists these domains and their respective clinical standards. A score of 0–100 is assigned to each domain. An average of the scores of the six domains for each NHS hospital is calculated to arrive at a composite measure of the clinical quality of the process of care for the hospital. We use the data for the 4-year period from the year 2004 to 2010 for English NHS trusts. It is clear from Fig. 1 that there is notable variation in the scores of hospitals that also changed over time. We conducted an analysis of variance, which shows that between-hospital variance (70.06) and within-hospital variance (42.77) account for 62 and 38% of the total variation in the data, respectively. Further information about the data, organisation, methods, proforma and questionnaire are available on https://www.rcplondon.ac.uk/.

### Organised stroke care

Stroke is a medical emergency that requires immediate medical intervention as the benefits are critically time dependent. To achieve this, a high level of coordination is required between various steps of care from the onset of stroke to rehabilitation. And this is only feasible if relevant complementary resources are in place and stroke specialists and trained staff collaborate with each other in a multidisciplinary and timely manner. To capture the organisational quality of the process of stroke care, the audit included a set of questions on the availability of stroke units, neurovascular facilities, TIA clinics and specialist stroke teams and their features, on whether the hospital provided education and engaged in clinical research, and on whether the hospital sought patient’s views and had produced reports within the last 12 months. The audit further collected data on average waiting time for scanning. Table 1 describes some of the hospital features about which the audit collected data. The NSSA summarises these features into eight organisational domains. They consist of acute care organisation, organisation of care, consultant sessions (overall service), interdisciplinary services (stroke unit), TIA/neurovascular service, education and research, team working and communication with patient and carers. On this basis, a composite measure of organisational quality is developed on a scale of 0 to 100 to assess the organisational quality of the stroke care process. The composite measure captures a mixture of organisational features and management practices critical for stroke care. Audit queries regarding the presence of a specialist stroke team or TIA clinic, for example, relate to the availability of resources. In contrast, audit queries relating to Stroke Unit Trialists’ Collaboration (STUC) characteristics, e.g., multidisciplinary meetings at least weekly to plan patient care, provision of information to patients about stroke or continuing education programmes for staff, or queries on whether patients’ views are sought refer to a management practices.

**Table 1:** Disaggregated variables for organisational design of stroke care

Variables	Description	Measure
ASU	Acute stroke unit	1 = yes; 0 otherwise
CSU	Combined stroke unit	1 = yes; 0 otherwise
RSU	Rehab stroke unit	1 = yes; 0 otherwise
Specialist	Presence of specialist stroke team	1 = yes; 0 otherwise
Report	Report produced with the last 12 months	1 = yes; 0 otherwise
PatientViews	Whether patient views were sought	1 = yes; 0 otherwise
SUTC	5 key features of all stroke units by Stroke Unit Trialists Collaboration	Integer scale from 1 to 5 where 5 if all features met
ESD	Presence of early supported discharge team	1 = yes; 0 otherwise
NeuroClinic	Presence of neurovascular/TIA clinic	1 = yes; 0 otherwsie

Over the years 2006–2010, the NSSA organisational audit went through changes in the form of inclusion of new and exclusion of old questions to collect data on a hospital’s organisational quality for stroke care. Despite the changes, a significant number of questions within the above domains remained the same. Particularly, the questionnaires used to collect data between 2006 and 2008 organisational audits were very similar or overlapping. We expect any impact of the organisational component on quality of stroke care to be slow. To capture these delayed effects we use 1-year lagged values of predictors when predicting the quality of stroke care instead of considering contemporaneous information. This leaves us with 2006 and 2008 organisational scores. Given the strong resemblance between 2006 and 2008 audits, we do not expect that the changes in the audits would distort our analysis. Further information on the data, questionnaire proforma and the method for constructing the composite organisational measure are available at https://www.rcplondon.ac.uk/ and in https://www.rcplondon.ac.uk/projects/national-sentinel-stroke-audit.

In addition to an aggregate measure, we supplement the analysis using disaggregated measures or factors of stroke organisation that are common across 2006 and 2008 and are listed in Table 1 above. These factors included in the audit are important markers for quality of care and capture aspects of organisational design for stroke care for each NHS hospital, i.e., their settings or configuration.

Table 2 below gives the descriptive statistics for the clinical and organisational quality of the process of stroke care for the NHS hospitals. Both the mean and median scores for clinical and organisational measures of the hospitals have been monotonically increasing over their respective sample periods. Conversely, the measure of variations captured by standard deviation for both the clinical process score and organisational performance has been decreasing except for an increase for process scores in 2006. Despite the gradual decline, variations in both clinical quality and organisational performance across the hospitals still persist. In other words, considerable heterogeneity exists with some hospitals providing better quality services than others.

**Table 2:** Descriptive statistics for clinical process score and organisational score

	Clinical process score				Organisational score		
	2004	2006	2008	2010	2006	2008	2010
Minimum	25.00	31.00	40.00	52.00	23.00	32.00	47.00
First quartile	51.75	58.25	63.00	73.00	57.25	63.75	62.00
Median	61.00	67.00	71.00	79.00	64.00	71.00	69.00
Mean	60.48	66.13	69.74	78.75	63.74	70.59	69.65
Third quartile	68.25	75.75	77.00	85.0	72.00	79.00	76.75
Maximum	93.00	93.00	96.00	97.00	89.00	95.00	96.00
Standard deviation	12.36	13.33	11.32	8.57	11.96	11.37	10.20

### Hospital and regional characteristics

We add several explanatory variables to our data to control for other factors that may affect health care quality. We add Teaching and Foundation Trust (FT) status for each hospital. Teaching hospitals tend to treat patients with the most severe and complex illnesses and have a higher chance of learning how to deal with complex cases. This may lead to a higher quality of care. Conversely, teaching hospitals can introduce a delay in the treatment process due to consultants’ role in training medical students [1].

Foundation trust hospitals are non-profit public organisations that enjoy greater managerial and financial autonomy from the central government control. FT hospitals are allowed to retain their surpluses, which they can invest in staff salaries or capital equipment. They are also allowed to borrow money to improve services. Hospitals with FT status are likely to have higher quality of care for stroke. Farrar et al. [23] find that FT hospitals provide a better quality of care measured using lower in-hospital mortality.

Larger NHS hospitals can lead to higher quality of care because of economies of scale and the “volume-outcome” hypothesis. Or, they might lead to lower quality of care due to diseconomies of scale from the greater complexity of their organisational structure. A number of studies include hospital size measured by the number of hospital beds among factors affecting hospital quality [40, 49, 62, 65]. We control for hospital size and case-load capacity using the number of hospital beds and the number of operating day-case theatres.

A rich literature highlights the importance of adequate specialists and neurologists in the prevention and management of stroke patients and improved stroke outcomes of care [17, 20, 53, 59, 66, 70, 73, 74, 78]. Hospitals with more skilled staff and specialists are likely to enjoy higher medical knowledge and experience and be capable of providing a relatively higher quality of stroke care. We include the number of neurologists, neurosurgeons and neurophysiology staff to control for the number of specialists. Park et al. [60] and Ketcham et al. [39] find that larger size physician groups are likely to improve health care quality. We add general measures of medical and non-medical staffs including professionally qualified staff and health care scientists.

A number of studies have found a positive relationship between nurse staffing and health care quality. Fine et al. [24] study the relationship between hospital characteristics such as nurse staffing and organisation type and measures of care for pneumonia. Raerty et al. [64] and Needleman et al. [56, 57] find a positive relationship between nurse staffing and health care quality in NHS and US hospitals. Cho and Yun [20] find that adequate nurse staffing measured by the bed-to-nurse ratio is positively associated with stroke care quality. We control for the number of nurses and the nurse-to-bed ratio.

Studies have also documented considerable regional variations in health care quality [32, 37, 45, 71]. Mooney [55] and Rudd et al. [69] find considerable variability in the quality of care for stroke across England. We consider median inflation-adjusted wage, inequality, the proportion of regional population without any qualifications, the number of stroke admissions, all-cause standardised mortality rate and the stroke mortality rate to control for socio-economic and regional-health factors. Gaynor et al. [29] control for regional measures including the median wage to proxy demand and need for care in a given area. Table 3 in the "Appendix" provides the full list of the variables used in the study, their definitions and sources.

## Panel regression trees

While the main objective of this article is to determine what predicts the quality of hospital care, our aim is to portray any predictor-response relationship in a simple, easy-to-understand and intuitive way. We are interested in making meaningful sense of how several predictors may be involved in complex interactions with one another when helping to explain quality. For example, we would like to understand whether the standard of care at hospitals that are located in a wealthy area and have a low nurse-to-bed ratio is different from the quality of care offered in hospitals with a higher nurse-to-bed ratio situated in a relatively lower income region. Traditional parametric methods such as regression models do not always offer straightforward interpretation of such intricate interplay of variables. We, therefore, resort to a class of non-parametric techniques, commonly known in machine learning literature as regression trees, that serve the purpose well. The tree mechanism involves recursively partitioning the predictor space into a number of small regions based on simple rules and then using the mean or median of the realised values (e.g., quality of care) of observations (e.g., hospitals) belonging to a region as the predicted value for a new observation that falls in that particular region. Most importantly, the splitting decision rules, order of importance of selected predictors and their interactions are summarised in a visually attractive and intuitive way. To our knowledge, this is one of the very few studies in health economics that exploit regression trees with an aim to find drivers of quality of care.

Several tree growing algorithms are proposed in the statistics and machine learning literature. The most widely used are ‘CART’ [13] and ‘C4.5’ [63], which function by maximising a statistical criterion over all possible predictors and split points simultaneously. These methods are often criticised for biased selection of variables, which have many possible splits and missing values [36]. In this article we opt to use a conditional inference framework proposed in Hothorn et al. [36] that rectifies the problem of selection bias by choosing predictors for splitting based on a series of tests identifying statistically significant association between the responses and predictors.

Since we observe a number of hospitals over several years the data used in our study are longitudinal or panel. Such a data structure requires careful accounting of possible variations across subjects that cannot be captured by observed predictors and also autocorrelation across observations from the same subject. If we observe subjects (e.g., hospitals) $i=1,2,\ldots ,I$ at times *t* (years) $=1,2,\ldots ,T$ a general additive model can be defined as:

1 $$\begin{aligned} y_{it} = f(\mathbf x _{it})+a_{i}+ u_{it} \end{aligned}$$

2 $$\begin{aligned} \left( \begin{array}{l} u_{i1}\\ \vdots \\ u_{iT} \end{array} \right) \sim N(0,R_i) \end{aligned}$$

3 $$\begin{aligned} a_i \sim N(0,D) \end{aligned}$$

where for each subject *i* and time period *t*, $y_{it}$ denotes the response of interest (e.g., quality of care), $\mathbf x _{it} = (x_{it1},\ldots ,x_{itK})^{'}$ denotes a vector of K predictors (e.g., hospital characteristics, regional features, etc.) and $u_{it}$ denote the errors. The $a_{i}$ are the time-invariant, subject-specific unobserved heterogeneity component. We allow for serial correlation in errors $u_{it}$ from a particular subject by defining their covariance matrix $R_i$ to be non-diagonal. The errors are, however, assumed to be independent across subjects and uncorrelated with $a_{i}$.

We seek to apply flexible tree-based methods for approximating the unknown relationship *f*, which may well be non-linear. It is only over the last decade that developments have been made in generalising trees for panel data applications. This article uses a method called an unbiased random effect expectation maximisation (RE-EM) tree, only recently developed in Fu and Simono [27]. Unlike many existing methods including a standard RE-EM tree of Sela and Simono [72], which rely on CART-type tree building algorithms, the unbiased RE-EM tree incorporates the conditional inference framework of Hothorn et al. [36] and is, therefore, unbiased in nominating predictors for splitting. The unbiased RE-EM tree operates by alternating between two principal steps: one estimating *f* by using a tree method and the other estimating unobserved heterogeneities $a_{i}$ by utilising a linear panel regression model. The process is initialised by setting the starting value of $a_{i}$ to zero.

As any non-parametric estimator, regression trees are subject to over-fitting. To achieve an optimal trade-off between the bias and variance (over-fitting), we rely on the out-of-sample predictive accuracy of the model, estimated using cross validation. We select the regression tree model with the lowest prediction error to identify variables that potentially contribute to the quality of stroke care.

By adopting the regression tree method, we complement the dominant tradition in econometrics, where the emphasis is on parameter estimation and statistical significance testing. There are no coefficients in regression trees. Instead, the idea is whether the variable of interest or the interactions implied by the background domain-specific information or theory appear in the tree that best predicts the outcome variable.

Our methodological stance comes close to the seminal work of Milton Friedman [26] and the practice in science: a minimum check for the empirical adequacy of a hypothesis is whether the predictions of the hypothesis are consistent with the empirical model that yields the best out-of-sample predictions. Such an approach does not establish the hypothesis. It can help narrow down the set of plausible hypotheses that are consistent with our data. Predictive consistency invites us to take a hypothesis seriously, assess its compatibility with our background knowledge and devise empirical studies that can support drawing causal conclusions.

## Empirical analysis

We employ an unbiased random regression tree estimator for panel data to develop a series of predictive models to identify key predictors of the quality of the process of stroke care in NHS hospitals. In building the tree models, we rely on the literature to decide on potential explanatory variables that may drive the quality of the process of stroke care across hospitals. Some of the potential control variables are highly correlated. The panel regression tree estimator arbitrarily selects from among highly correlated variables to build a model [41]. To gain insight into the possible impact of each variable, we use a data-driven method proposed in Kuhn and Johnson [41] to a priori select from among the correlated variables to build a model and further examine the effect of replacing the variables with excluded variables to assess the robustness of the results. (The supplementary results are available on request.) Two variables are considered as highly correlated when the correlation coefficient exceeds the threshold 0.75. All unbiased REEM-Tree models are fitted using a random intercept model to allow for variations in hospitals' quality due to unobserved attributes.

Among the variables measuring physical capital, the variables operating theatres and day case theatres are highly correlated (0.88). We a priori exclude the number of operating theatres from the base model. The correlation among nurses, general medicine group (gmg) staff, science and allied professionals staff exceeds the threshold of 0.75. We keep nurses in the base model, partly because this choice will lead to models with overall lower out-of-sample prediction error. The number of nurses in a hospital is highly correlated with the number of beds. We replace nurses with the nurse-to-bed ratio in order to be able to identify possible distinct effects of physical capital (or hospital size) and non-specialist human capital. Among the regional health and socio-economic variables, the weekly median wage and inequality are highly correlated (0.845). We exclude inequality from the base models. Also, stroke mortality and the all-age standardised mortality ratio (SMR) are highly correlated (0.87). We keep stroke mortality.

We consider four unbiased regression tree models to gain insights into the structure of the data. The first three models regress the measure of the quality of the clinical process of stroke care on the individual variables that capture organisational features of the process of stroke services and relevant control variables. The fourth regression tree model replaces the individual variables with the composite measure of organisational quality of the process of stroke care to better capture possible complementarity.

We next compare the results of the unbiased REEM-Tree with linear mixed effects models (LME) to check the robustness of the results. We rely on the in-sample and out-of-sample predictive performance of the models to select a model from among the trees that best fits the data.

### Disaggregate models

Recall we categorised possible drivers of the quality of stroke care into internal and external drivers of hospital performance. Internal drivers refer to factors such as physical assets, human capital, the structure of decisions and the internal organisation of the hospital. External drivers refer to regional health and socio-economic features. We start with a model of internal drivers and next adds variables capturing potential external drivers. Our underlying methodological principle is that if any of these factors drive the quality of stroke care, variables measuring the factors will appear in a model that best predicts the quality of stroke care [26]. With these preliminaries, the first panel regression tree model includes the primary organisational variables and the variables measuring physical assets, human capital and hospital characteristics. This means the variables entering the formula underlying the model consist of:

**Model 1:** *Score* $\sim$ *beds + day case theatres + nurses + neurology + neurophysiology + neurosurgeons + teaching + ft + ASU + RSU + CSU + SUTC + Specialist + ESD + NeuroClinic + PatientViews + Report*.

**Fig. 2:**
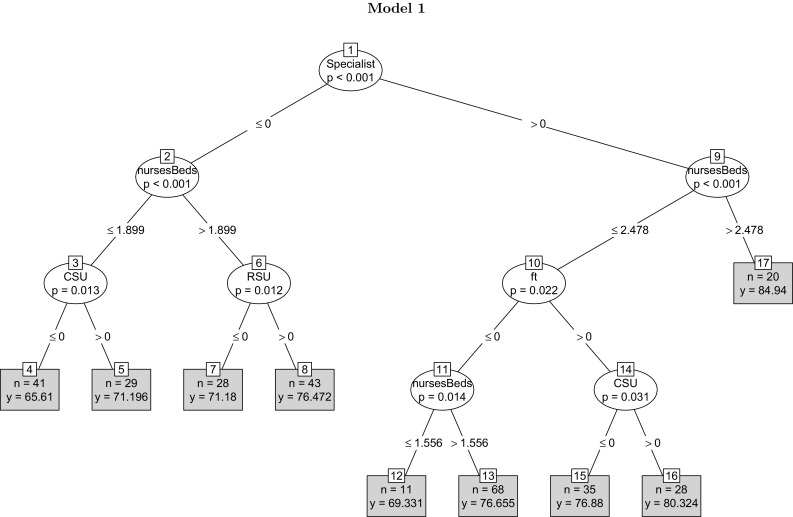
The tree includes variables capturing hospital characteristics, human capital factors, teaching and foundation trust status, and primary variables reflecting the quality of the organisation of the process of stroke care. The higher a variable appears in the tree structure, the more predictively significant the variable will be. The organisational variable ‘Specialist’ appears at the initial node of the tree as the predictively most important variable. Each *box* in the terminal nodes show two figures, the first (*n*) stating the number of observations falling in the branch and the second (*y*) giving the mean value of the observations in the branch. Hospitals with the highest quality score fall in the branch where the value of the dummy "specialist" equals one and the nurse-to-bed ratio exceeds 2.478

The dependent variable *score* measures the quality of the clinical process of stroke care. Table 3 defines the rest of the variables. Applying the unbiased panel regression tree estimator to the data yields the tree in Fig. 2. For the current analysis, a significance threshold of 0.10 for unbiased REEM-Trees has been set (i.e., a 10% false-positive rate for statistical significance is set). The variable ‘specialist’ appears in the initial node of the tree, suggesting that the presence of a specialist stroke team is the most important predictor of the quality of stroke care. Hospitals with specialist stroke teams enjoy an overall higher quality score, which is in line with the findings in Xian et al. [80]. Indeed, the highest quality score belongs to hospitals with a specialist stroke team and a higher nurse-to-bed ratio (with mean quality score 84.94), confirming the findings in Cho and Yun [20]. Also, as found in Farrar et al. [23], among hospitals with a specialist stroke team, foundation trust hospitals yield a higher quality score. The lowest quality score appears in hospitals that lack a specialist stroke team, have a low nurse-to-bed ratio and lack a combined stroke unit (CSU) (with the mean quality being 65.61) [16]. Overall, the model points to the significance of specialist human capital and organisational variables. The variables representing physical capital do not appear in the unbiased panel tree. The model points to critical complementarities between the presence of a specialist stroke team and a high nurse-to-bed ratio. It is the joint presence of these features that predicts a higher quality.

Adding the variables representing potential external drivers of quality will give rise to our second model:

**Model 2:** *Score* $\sim$ *beds + day case theatres + nurses + neurology + neurophysiology + neurosurgeons + teaching hospital + foundation trust + ASU + RSU + CSU + SUTC + Specialist + ESD + NeuroClinic + PatientViews + Report + stroke admissions + stroke mortality + median wage + no qualifications*.

**Fig. 3:**
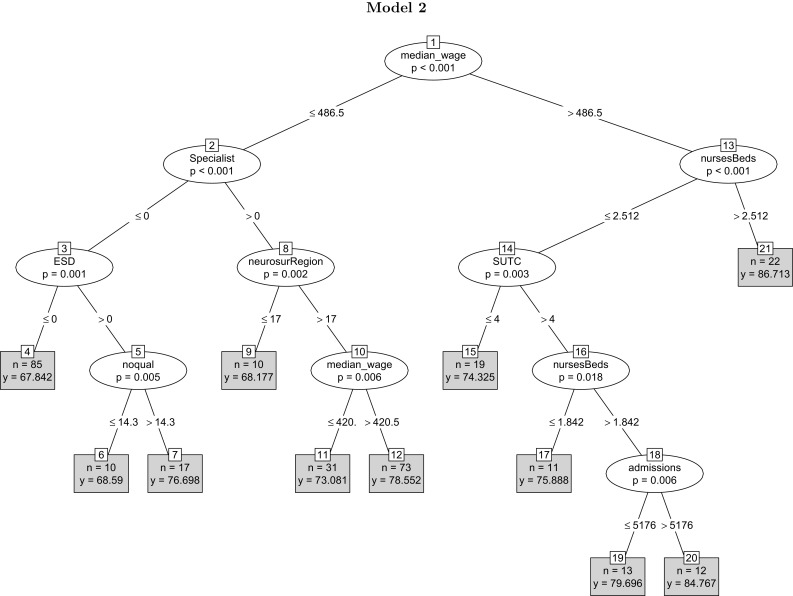
The tree adds external variables such as weekly median wage to the variables underlying model 1. Weekly median wage appears at the start of the tree as the predictively most significant variable, followed by the nurse-to-bed ratio and specialist. Other variables measuring the organisational feature of the process of stroke care occupy important positions in the tree, supporting the idea that organisational factors are among the most significant predictors of the clinical quality of care

From the tree results in Fig. 3, median wage appears at the initial node of the tree, suggesting that economic conditions are among important predictors of the quality of stroke care [37]. The mean quality of stroke care in areas with a higher weekly median wage (>486.5) is on average higher. On the right-hand-side branch, the second predictively most significant variable is the nurse-to-bed ratio. The equality of the process of stroke care is the highest in hospitals in affluent areas where the nurse-to-bed ratio exceeds (2.512) [12]. On the left-hand side, specialist is the second predictively most important variable. The quality of the process of stroke care is overall higher in hospitals with a specialist stroke team in place. The variables appearing in the third layer of the tree include those that capture the organisation of the process of stroke care. The presence of a stroke specialist team, SUTC, and the presence of an early support discharge team (ESD) occupy prominent positions in the tree [43, 67]. The tree also reveals important interactions (complementarities) among the variables. The interaction between a comparatively lower median wage (<486.5) and the absence of a stroke specialist team where there is no early support discharge team (ESD) yields the lowest mean average quality score.

Overall, the model points to several results. To begin with, the model reveals variations in the quality of stroke care across regions, consistent with the findings in Grimaud et al. [33]. An explanation for the regional variation is that wealthier regions have more access to higher quality human capital resources, which is likely to enhance the quality of care a hospital provides. Another explanation, which receives support from our data, is that hospitals in richer regions devote more resources to health care [21]. In the sample, the median wage is reasonably highly correlated with the nurse staff ratio (0.437), clinical staff ratio (0.47) and gmg staff ratio (0.404). Median wage is also negatively correlated with regional stroke mortality. A third explanation is that a higher median wage is associated with higher education [37]. Patients with higher levels of education are likely to be aware of their rights and the quality of services they receive. And this can translate into pressures on hospitals to provide higher quality services. The model also points to the pivotal role of the organisational features of the stroke care process in driving the quality of stroke care. The organisational variables occupy prominent positions in the regression tree. Finally, the model casts doubt on whether hospital size affects the quality of the process of stroke care. The number of beds, which measures hospital size, does not appear in the tree. Any claim for the causal validity of these results call for further analysis.

Figure 1 previously revealed a shift in the distribution of the stroke quality measure over the sample period. The monotonic increase in the mean quality score could be due to a change in the underlying joint probability distribution of the variables driving the quality of stroke care. We next include year dummies in the last model to capture the time trend and examine whether the key patterns observed in the trees remain unchanged. Our sample spans over four periods from year 2004 to year 2010. However, the data on the organisational variables are not available for year 2004. Further, the predictor variables have been lagged to deal with contemporaneous endogeneity. This means there are effectively data for two periods in the unbiased tree estimation. We take the year 2008 as the base year and introduce a dummy variable ‘yr2010’ that takes value 1 if an observation belongs to year 2010 and zero otherwise. Adding the dummy variable to the list of variables will lead to our third model:

**Model 3:** *Score* $\sim$ *beds + day case theatres + nurses + neurology + neurophysiology + neurosurgeons + teaching hospital + ft + ASU + RSU + CSU + SUTC + Specialist + ESD + NeuroClinic + PatientViews + Report + stroke admissions + stroke mortality + median wage + no qualifications + yr2010*.

**Fig. 4:**
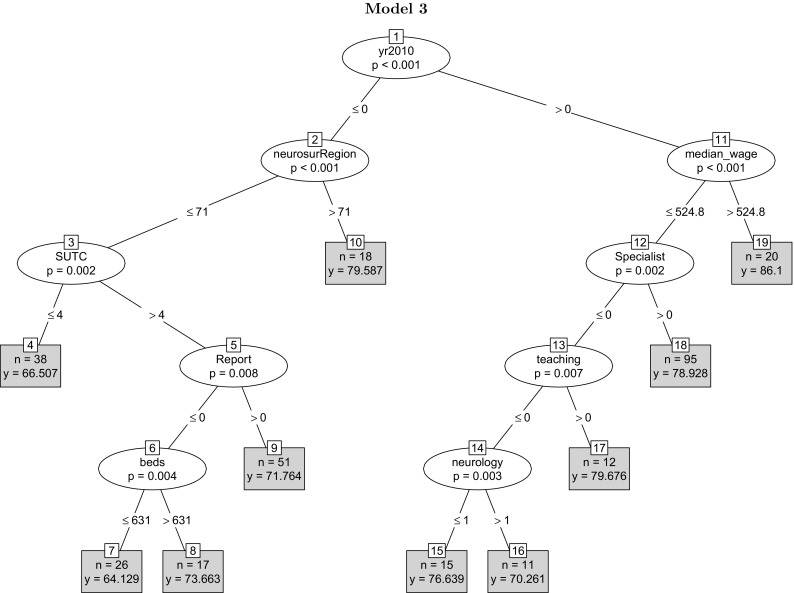
The tree adds a year dummy to the variables in tree model 2. The dummy variable for year 2010 appears at the initial node of the tree as the most predictively significant factor, followed by median weekly wage and neurosurgeon per region. The mean quality score is higher in year 2010, consistent with the descriptive results in Fig. 1. The variables reflecting the organisational quality of stroke care occupy prominent positions. A number of variables including ‘day case theatres’, ‘neurology’ and FT fails to appear in the tree

In Fig. 4 the year dummy appears in the initial node of the tree, indicating a higher mean quality of stroke care in year 2010. A reason for the split of the initial node at 2010 can be the implementation of the National Stroke Strategy Policy in NHS England, which was implemented in December 2007. The policy measures included monitoring and assessing the quality of stroke care across NHS hospitals, which might have shifted the distribution of the overall measure of the quality of stroke care. The second set of most predictively significant variables are weekly median wage and neurosurgeons per region. The average clinical quality of stroke care is higher in wealthier regions or regions with a higher number of neurosurgeons. Considering the fact that only a small proportion of stroke patients requires surgery we take caution in interpreting the relevance of the number of neurosurgeons by identifying it as an indicator of broader specialist capacity rather than an absolute/direct driver of quality of stroke care in hospitals. Primary organisational variables occupy the next prominent positions in the tree. Consistent with the previous results, higher clinical quality of stroke care is correlated with better organisation of stroke care. The presence of the organisational score variable is robust to including year as a predictor.

### Composite measure

A fundamental insight of economic theory is the critical complementarity among diverse elements that shape the organisation of the firm and its performance [14]. In Fig. 2, in the left-hand-side branch, the absence of a specialist stroke team, a comparatively lower nurse-to-bed ratio and the absence of a combined stroke unit jointly give rise to a lower mean average quality of stroke care, or having a stroke specialist team present in a foundation trust hospital leads to higher mean stroke care quality. To capture complementarities among organisational factors contributing to the process of stroke care, we adopt a composite measure of overall organisation of the process of stroke care constructed in the National Sentinel Stroke Audit (NSSA). Replacing the disaggregate indicators of organisational quality with the composite measure, which is derived from the primary indicators, while maintaining other variables in the model, will yield the model:

**Model 4:** *Score* $\sim$ *beds + day case theatres + nurses + neurology + neurophysiology + neurosurgery + teaching + ft + admissions + stroke mortality + median wage + no qualifications + organisation score + yr2010*.

**Fig. 5:**
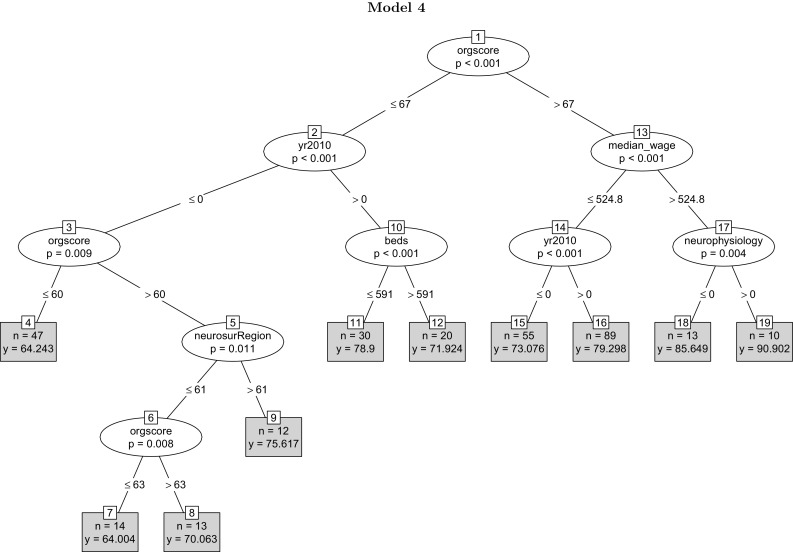
The unbiased tree replaces the composite organisational measure for the primary features of the organisation of the process of stroke care. The variable appears as the most predictively significant factor, followed by median weekly wage and the year dummy. The highest quality score belongs to hospitals with a high organisational score and with the neurophysiology variable taking a value located in wealthier regions. Variables day case theatres, nurse-to-bed, neurology, neurosurgery, teaching, FT, admissions, stork mortality and ‘no qualifications’ fail to appear in the tree

Figure 5 represents the unbiased panel regression tree built from these variables. The composite measure of the organisational quality appears at the initial node of the tree, indicating the organisational quality of the process of stroke care as the most prominent predictor of the clinical quality of stroke care. Hospitals with high organisational quality (>67) located in comparatively affluent areas enjoy the highest quality of stroke care. The mean predicted scores for year 2010 wherever the year dummy appears are higher than the mean predicted scores for year 2008. Further, but interestingly, hospitals with a low composite organisation score (<67) but a larger number of beds (>591) reveal a relatively lower mean quality of stroke care. In the absence of proper organisation of resources, larger hospitals might be at a disadvantage. Finally, the lowest mean quality of stroke care belongs to hospitals in regions with a comparatively lower number of neurosurgeons (<61) and low composite organisation score (<64). The reason for the relative simplicity of the model is manifold: Human capital variables strongly predict the organisation score; weekly median wage is also highly correlated with other variables capturing the health and socio-economic regional characteristics; human capital variables are highly correlated among themselves and with variables such as (the number of) beds. Overall, once the composite measure of organisation quality of the process of care is included among the explanatory variables, it appears as the predictively most significant variable, consistent with the recent economic research on productivity that identifies organisational features and management practices and style as the deepest drivers of firm performance. The figures highlight the importance of complementarity as in Milgrom and Roberts [52] and Brynjolfsson and Milgrom [14]. The results of the tree models confirm the insights of the wider economics productivity literature on the importance of the organisation of resources, organisational design and management practices.

Table 5 reports the in-sample goodness of fit measures AIC and BIC for the four unbiased regression tree models. The in-sample fit measure AIC and BIC scores monotonically decrease from model 1 (2195.83) to model 4 (2096.64), suggesting that the unbiased regression tree model 4, with the composite organisation measure, fits the data best. According to Burnham and Anderson [15], differences in the values of AIC of around 4–7 correspond to roughly 95% significance. The table also reports the leave-one-out and *k*-fold out-of-sample-predictive performance measures for the unbiased tree models. Consistent with the in-sample fit measures, the final model yields the lowest out-of-sample prediction error among the models—(8.531) and (8.268) respectively.

**Fig. 6:**
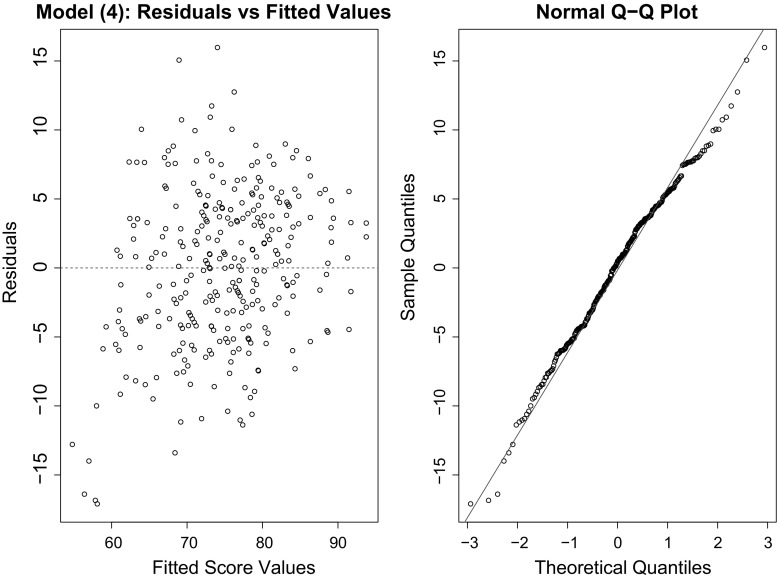
In-sample fit plot for the unbiased tree for model 5

Figure 6 provides the diagnostic plots for unbiased REEM-Tree model 4. The residuals from the model are approximately normally distributed, as indicated from the Normal Q–Q plot in the left panel of the figure. The right panel of the figure gives the residuals versus fitted values plot to check for constant variance or homoscedasticity assumption of errors. The residuals are scattered around the horizontal line and the width of the data points are equal throughout, confirming homoscedasticity. The model enjoys a satisfactory in-sample goodness of fit.

## Robustness analysis

The regression trees presented above do not take care of hospital fixed effects features. In the absence of strong theory, one requires alternative techniques to capture possible causal connections. We build a series of linear panel data models to investigate whether the coefficients of the variables appearing in the trees are statistically significant and whether the coefficient estimates are robust to the inclusion of hospital individual fixed effects. Further, we report whether the results are robust to the choice of alternative measures of physical capital, human capital and regional factors. We also consider replacing our human capital variables with relevant ratios.

### Correlated random effects models

We first check the empirical validity of the patterns observed in trees by comparing them to a class of linear mixed effects models (LME). The LME results come quite close to traditional random-effects results. Both assume that $a_{i}$ in Eq. (1) uncorrelated with explanatory variables. We have no theoretical ground to warrant such an assumption. A prime reason for choosing LME as a benchmark is its consistency with the REEMtree. The unbiased panel tree estimator is built around LME. Further, our interest is to estimate possible impacts of time invariant variables such as the teaching or foundation trust status of hospitals on quality of care, which makes a standard fixed effect (FE) estimator unsuitable. Next we estimate a correlated random effects (CRE) model that is a flexible extension to RE and is comparable to FE [79]. Consider the simple case of the panel model in Eq. (1), with a single time-varying explanatory variable $x_{it}$. $a_{i}$ may be correlated with $\{x_{it}: t = 1, 2, \ldots , T\}$.

4 $$\begin{aligned} y_{it} =\beta _{i}x_{it} + a_{i} + u_{it}, \quad t=1, 2, \ldots , T. \end{aligned}$$

In CRE, we model the correlation between $a_{i}$ and $\{x_{it}: t = 1, 2, \ldots , T\}$. Since $a_{i}$ is, by definition, constant over time, it can be correlated with the average level of the $x_{it}$. More specifically, let $\bar{x}_{i} = T^{-1}\Sigma ^{t}_{i =1}x_{it}$ be the time average. It is plausible to assume the simple linear relationship

5 $$\begin{aligned} a_{i} = \alpha + \lambda \bar{x}_{i} + r_{i} \end{aligned}$$

where $r_{i}$, by assumption, is uncorrelated with each $x_{it}$. The correlated random effects approach uses (5) in conjunction with (4). Substituting the former into the latter gives:

6 $$\begin{aligned} y_{it} =\beta _{i}x_{it} + \alpha + \lambda \bar{x}_{i} + r_{i} + u_{it} = \alpha + \beta _{i}x_{it} + \lambda \bar{x}_{i} + r_{i} + u_{it} \end{aligned}$$

The equation has a composite error term $r_{i} + u_{it}$, consisting of a time-invariant unobservable $r_{i}$ and the idiosyncratic shocks, $u_{it}$. Further, because $u_{it}$ is assumed to be uncorrelated with $x_{it}$, all *s* and *t*, $u_{it}$ is uncorrelated with $\bar{x}_{i}$. With these assumptions, the estimation problem becomes identical to the random effects estimation of

7 $$\begin{aligned} y_{it} = \alpha + \beta _{i}x_{it} + \lambda \bar{x}_{i} + r_{i} + u_{it}. \end{aligned}$$

The addition of $\bar{x}_{i}$ controls for the correlation between $a_{i}$ and the sequence $\{x_{it}: t = 1, 2, \ldots , T\}$. Wooldridge (2010, Chapter 10) shows that $\hat{\beta }_{CRE} = \hat{\beta }_{FE}$, where $\hat{\beta }_{FE}$ stands for the fixed effects estimator. The CRE approach, thus, provides a way to include time-invariant explanatory variables in what is effectively a fixed effects analysis.

We assess the robustness of the four models introduced earlier. In each case, we consider two LME models: (1) a model with all the variables entering the corresponding tree and (2) a model with the within-group means of the time-varying variables to capture fixed effects.

Table 6 presents the first set of the LME results. Each column belongs to the four models respectively. The first column includes the internal drivers of quality of stroke care entering unbiased regression tree model 1 along with the dis-aggregate measures of organisational performance. Consistent with the tree model 1, variables nurse-to-bed ratio (5.490), specialist stroke team (5.172), foundation trust (2.487) and CSU (3.795) all are statistically significant at less than the 5% critical level. In addition, the variable SUTC (2.461) is also statistically significant at less than the 5% level. These predictors have material effects on the quality of stroke care. For example, a unit change in the nurse-to-bed ratio results in a 5.49-unit change in the quality of care index, given other variables are held constant. The quality of care in a hospital that has a Specialist Stroke Team is 5.172 points higher than a hospital that does not have such a specialist team. Presence of a Combined Stroke Unit (CSU) is associated with a 3.795-point improvement in quality while a foundation trust status accounts for an additional 2.461 points. Column 2 corresponds to regression tree model 2, which adds potential external drivers of the quality of stroke care to the variables in the first model. Variables median wage (0.084), NeuroClinic (4.709), STUC (2.639), specialist (3.935) and foundation trust (3.332) are statistically significant at less than the 5% level. Presence of a Neuroclinic improves the quality score by 4.709 units. Foundation trust status, provision for a specialist stroke team and availability of well-equipped stroke units (SUTC) all maintain sizeable effects on quality as before with coefficients of 3.332, 3.935 and 2.639, respectively, but the impact of median wage is small (0.084). There are discrepancies between the regression tree results and the LME results—notwithstanding, the organisational variables appear prominent in both LME models. Variables Specialist and SUTC, which are significant in both regression models, occupy prominent positions in the trees too. Column 3 adds the year dummy to the list of the variables. With the inclusion of the year dummy, median wage is no longer statistically significant. In addition to the year dummy (6.574), SUTC (2.748), neuroclinc (3.437) and neurosurgeon per region (0.047) are all statistically significant at less than the 5% level. The quality score was on average 6.574 points higher in the year 2010 compared to the year 2008. This implies that the Department of Health’s National Stroke Strategy, which was implemented across English NHS trusts in December 2007, resulted in profound and significant improvement in hospital performance. Importantly, though, even after controlling for the year effect, the effects of some stroke care resources, such as SUTC and Neuroclinic, remain considerably large (2.748 and 3.437, respectively). As for regression tree results, we define neurosurgeons per region as a wider measure of specialist input when interpreting its importance in predicting quality of stroke care. Column 4 replaces the primary organisational features of the process of stroke care with the composite measure of organisational quality of the process of stroke care. With the composite measure included, variables FT (foundation trust status) (1.824), stroke mortality (0.230), median wage (0.057), the year dummy (4.831) and organisation score (0.331) are all statistically significant at less than the 5% level. The fact that the composite organisational score has a small effect (0.331) on stroke quality possibly suggests that individual components have disproportionate, and in certain cases opposite, effects on quality. Overall, if any lesson can be drawn from these results, it is the conclusion that organisation of the process of stroke care matters greatly. The organisational variables in one form or another appear prominent in both estimation approaches.

The Hausman test indicates the presence of fixed effects. Table 7 represents the LME results when, in line with the correlated random effects approach, we add the within-group means of the time-varying variables to the list of the variables to capture potential fixed effects. In column 1, we add the within-group means of the time-varying variables to the list of the variables in model 1 that only includes potential primary internal drivers of the clinical quality of the process of stroke care. The fixed effects coefficients of the key organisational variables CSU (3.716), SUTC (2.389), specialist (5.394) and foundation trust (2.614) are all statistically significant at 5% or below. No other variables appear as statistically significant. Column 2 adds the within-group means of the time-varying external drivers of the quality score. The organisational variables SUTC (2.559), specialist (2.575) and NeuroClinic (4.069) are statistically significant at the 5% level or below. The variable stroke admissions is significant at below the 5% level with fixed effect coefficient 0.030. Mean admissions ($-0.030$) and stroke mortality (1.715) are significant at the $5\%$ level. Column 3 adds the year dummy variable. The variable is significant at the $5\%$ level, with a high magnitude (11.414), emphasising again the important impact of the policy change mentioned before. Importantly, the organisational variables SUTC (2.58) and specialist (2.308) remain statistically significant at the 5% level or below. Column 4 replaces the primary organisational variables in model 3 with the composite organisational measure. Besides the year dummy (10.570), the mean of the composite measure appears statistically significant (0.449) at below the 1% level. All in all, even when we consider fixed effects coefficient estimates, the variables capturing aspects of the organisational quality of the process of stroke care such as SUTC remain significant. The fixed effects coefficients of the rest of the variables are not statistically significant.

In the above, we did not separate hospital fixed effects from time fixed effects. Testing for hospital and time effects, there is strong evidence for the presence of time effects, which is consistent with the observed pattern in Fig. 1. We turn to conventional panel regression to examine time fixed effects separately. Table 8 shows three models with time effects. Column 1 presents the results for the model with the internal drivers of clinical quality of the process of stroke care, using disaggregated organisational measures. Column 2 adds potential external drivers. Column 3 replaces the disaggregate organisational indicators with the composite measure of organisational quality of the process of stroke care. The disaggregate organisational variables SUTC and specialist are statistically significant in the first two columns. The variable neurology in the first model and NeuroClinc (the presence of a neuro clinic) in the second model are statistically significant too. And the composite measure of organisational quality of the process of stroke care appears strongly statistically significant in the third model too. All the three models point to the statistical significance of organisational features of the process of stroke care. [The presence of time effects points to the possibility that changes in the environment (say policies) may have affected the quality of stroke care, but in the same way for all the hospitals.]

Further, the nurse-to-bed ratio variable appears statistically significant in the first two models, with a positive sign across all the models, suggesting complementarity between the number of nurses and beds. The coefficient for beds, as a measure of hospital size, has a negative sign across the models and is significant only in the first model. The measures indicating external factors are statistically significant in one form or another. The number of neurosurgeons per region is statistically and positively significant in the second model and weekly median wage in the third model. The variable stroke mortality is statistically significant in both models—suggesting that in areas where stroke mortality increased over time the quality of the process of stroke care has improved too. It is possible that extra resources are devoted to the process of stroke care in areas with high stroke mortality, leading to a higher correlation between stroke mortality and the clinical quality of stroke care.

To further support the robustness of our key results, we rescale the measures of the quality of the clinical and organisation of the process of stroke care by taking out the within-hospital means of the variables, while including the control variables in their level form. Table 9 reports the results with the transformed variables. Column 2 adds median wage to the list of control variables in column 1, column 3 excludes the data on London hospitals, and column 4 adds London to the list of control variables. In all the columns, the coefficient estimate of the composite organisational score, in its transformed form, is significant at 1%, with the magnitude ranging from 0.297 to 0.326. Changes in the quality of the clinical process of stroke care are strongly positively associated with changes in the composite organisational score. And the coefficient of the year dummy is no longer statistically significant. The results are consistent with the theoretically plausible conjecture that variations in the quality of the organisation of the process of care drives changes in the quality of the process of stroke care. While the panel structure of the short sample supports our hypothesis, there is no claim for causation. There could be unobserved time-varying factors that drive changes in both clinical and organisational measures of quality.

The fact that the organisational quality of the process of stroke care appears among critical drivers of the clinical quality of stroke care raises a deeper question as to what factors drive organisational quality. Our sample is inadequate for fully shedding light on the subject. Nonetheless, we have regressed the composite measure of organisational quality on several theoretically plausible drivers of organisational quality. Columns 1 and 2 in Table 10 include several internal drivers. Column 1 controls for individual fixed effects and column 2 controls for both individual and time fixed effects. Columns 3 and 4 add weekly median wage. The columns respectively control for individual fixed effects and both individual and time fixed effects. In all the columns, the nurse-to-bed ratio is statistically and quantitatively significant at the (10%) level or below. The median weekly wage appears as statistically significant in the last column, suggesting that hospitals located in more affluent areas have higher organisational quality.

The results from the traditional econometric approaches corroborate the findings from the unbiased REEM-Trees. All the analyses indicate the importance of the organisational quality of stroke care in determining the clinical quality of stroke care. The tree models point to critical interactions among various variables driving stroke care quality. It is a complex interaction among hospital resources that shapes quality. Our key results are robust to the use of ratio variables or alternative measures of human or physical capital left out in constructing the trees because of the high correlation. Our results are not driven by hospitals in London. Excluding data relating to London’s hospitals will not change our results, as shown in Table 9.

## Conclusion

A feature of the health care systems is substantial variation in health care quality across hospitals. This study has focused on the quality of the process of stroke care as an indicator of the overall quality of NHS hospitals. Drawing on the literature, we conjectured several explanations for the observed cross-hospital heterogeneity and tested their predictive implications using a short panel spanning 2004 to 2010. Both the parametric and non-parametric methods identify several features of the organisation of stroke care as key predictors of hospital quality. Presences of specialist stroke teams, well-equipped stroke units and neurovascular clinics have all been found to improve the quality of stroke care and the improvements range between 2 to 5 points in a quality score measured on a 0–100 scale. When a composite score as a summary of such organisation of care has been used separately it consistently appeared to be a significantly important driver of quality. The result persists when potential confounders/variables are controlled for and is particularly robust to the inclusion of a time trend, which accounted for a major policy change during the study period. The non-parametric methods identify the measure of organisation score as the key predictor of hospital quality. The result persists when potential confounders/variables are controlled for and is particularly robust to the inclusion of a time trend. The result supports an emerging literature that emphasises the critical role of organisation of resources in productivity. The non-parametric methods also reveal critical interactions among potential determinants of the quality of the process of stroke care.

Several limitations offer scope for further research. The predictive results narrow down candidate hypotheses on the sources of hospital heterogeneity but are only indicative of the causal impact of organisational factors on the quality of stroke care; they do not establish causation. A thorough analysis will consider further control variables such as stroke admissions, examine alternative measures of quality and attempt to obtain a more comprehensive measure of organisation quality. As another limitation, our study does not control for clinical networks. Under these clinical networks, hospitals combine and integrate resources with other hospitals in the networks to provide high quality of care to the patients. As mentioned in [61], for example, cardiac networks in the English NHS have led to an increase in the rate of specialist interventions and faster access time. The results trace differences in the quality of the process of care to differences in the organisation of resources and management practices across NHS hospitals. Ultimately, it is vital to explain organisational heterogeneity—why some hospitals enjoy a higher level of organisational design than others. Future research will benefit from using a longer longitudinal framework to better identify trends in performance. Equally, it will prove insightful to compare the effects of the National Stroke Strategy that was implemented in England and compare the quality for stroke care with Wales and Northern Ireland. This allows one to draw causal effects of such policy changes (using quasi-experimental techniques such as difference in difference) since health care systems across England and in Wales or Northern Ireland are broadly comparable. Finally, future research needs to take into account detailed staff data related to stroke care including rehabilitation and geriatric staff and control for cardiac networks.

## Appendix

See Tables 3, 4, 5, 6, 7, 8, 9 and 10

**Table 3:** Definition and source of variables used in the analysis

Variable	Definition	Source
Clinical process of care		
score	Total clinical process score	The National Sentinel
		Stroke Audit; RCP
Organisational variables		
Organisation score	Total organisational score	The National Sentinel
		Stroke Audit; RCP
Hospital characteristics		
Teaching hospital	Whether the hospital is a teaching hospital	Self-coded from hospital’s website
Foundation trust	Whether the hospital is a foundation trust	Self-coded from Monitor website
Human capital		
Non-medical staff	Headcount of non-medical staff as of 30 September	NHS workforce; statistics, (HCHS)
Scientific staff	All qualified scientific, therapeutic and technical staff as of 30 September	NHS workforce; statistics (HCHS)
Allied professionals	Qualified allied health professionals as of 30 September	NHS workforce; statistics, (HCHS)
Clinical staff	Total number of medical and dental staff as of 30 September	NHS workforce; statistics, (HCHS)
Qualified clinical staff	Headcount of professionally qualified clinical staff as of 30 September	NHS workforce statistics (HCHS)
Nurses	Headcount of qualified nursing midwifery and health visiting staff as of 30 September	NHS workforce statistics (HCHS)
General Medicine Group	Total medical staff at General Medicine Group as of 30 September	NHS workforce statistics (HCHS)
Neurology	Total medical staff at Neurology group as of 30 September	NHS workforce; statistics, (HCHS)
Neurophysiology	Number of medical staff at clinical neurophysiology group as of 30 September	NHS workforce statistics (HCHS)
Neurosurgeons	Number of neurosurgeons as of 30 September	NHS workforce statistics (HCHS)
Physical capital		
Beds	Total number of available beds	Hospital Estate and facilities data
Operating theatres	Number of operating theatres, quarter ending September	Dept. of Health QMCO
Day case theatres	Number of dedicated day case theatres quarter ending September	Dept. of Health QMCO
Regional characteristics		
Stroke admissions	Regional emergency stroke admissions standardised by age and gender	HSCIC
Stroke mortality	Regional stroke mortality standardised by age and gender	HSCIC
All SMR	All age standardised mortality ratio (SMR)	HSCIC
Median wage	Regional full-time weekly median wage	ONS; ASHE
Inequality	Ratio of 10th and 90th percentile full time weekly wage	ONS; ASHE
No qualifications	Regional population with no qualifications (%)	NOMIS

**Table 4:** Clinical process score domains

Clinical domains	Clinical standards
Initial patient assessment	Screening for swallowing 24 h
	Visual fields
	Sensory testing
	Brain scan within 24 h of stroke
Multidisciplinary assessment	Swallowing assessment speech and language therapist
	Physiotherapy assessment within 72 h
	Initial assessment of communication 7 days
	Occupational therapy assessment within 4 working days
	Social work assessment within 7 days of referral
Screening and functional assessment	Patient weighed at least once during admission
	Evidence mood assessed
	Cognitive status assessed
	Screening for malnutrition
Care planning	Evidence of rehabilitation goals
	Plan to promote urinary continence
	Receiving nutrition within 72 h
Communication with patients and carers	Discussion with patient about diagnosis
	Carer needs for support assessed separately
	Skills taught to care for patient at home
	Follow-up appointment at 6 weeks
	Driving advice
Acute care	Aspirin within 48 h of stroke
	90% of stay in a sroke unit
	Admitted to an acute or combined Stroke Unit within 4 h
	Receiving fluids within 24 h
	Receiving thrombolysis

**Table 5:** In- and out-sample fit measures

	In-sample fit		Out-sample fit	
	AIC	BIC	LOOCV	*k*-fold
	(1)	(2)	(3)	(4)
Models				
Model 1	2195.83	2236.35	9.314	9.319
Model 2	2147.44	2195.23	9.204	9.091
Model 3	2131.69	2175.86	8.562	8.405
Model 4	2096.64	2140.80	8.531	8.268

**Table 6:** Linear mixed effects regression results

	Models			
	(1)	(2)	(3)	(4)
(Intercept)	44.361 (5.706)***	−7.571 (23.684)	14.724 (23.185)	−4.762 (21.197)
Beds	−0.003 (0.002)	−0.000 (0.002)	−0.002 (0.002)	−0.002 (0.002)
Day case theatres	−0.117 (0.263)	−0.371 (0.264)	−0.172 (0.262)	−0.231 (0.236)
Nurse-to-bed ratio	5.490 (1.567)***	2.175 (1.701)	2.259 (1.648)	1.916 (1.525)
Neurology	0.168 (0.108)	0.015 (0.110)	0.067 (0.109)	0.036 (0.097)
Neurophysiology	0.311 (0.650)	0.340 (0.649)	0.392 (0.638)	0.291 (0.579)
Neurosurgery	−0.087 (0.147)	0.001 (0.145)	−0.019 (0.143)	−0.026 (0.128)
Teaching	1.849 (1.549)	1.580 (1.533)	1.555 (1.504)	1.164 (1.331)
Foundation trust	2.487 (1.226)**	3.332 (1.209)***	1.445 (1.223)	1.884 (1.117)*
ASU	1.524 (1.937)	1.576 (1.875)	−0.370 (1.830)	
RSU	1.066 (1.761)	0.388 (1.739)	0.625 (1.676)	
CSU	3.795 (2.181)*	2.172 (2.145)	0.129 (2.100)	
SUTC	2.461 (1.011)**	2.639 (0.977)***	2.748 (0.935)***	
Specialist	5.172 (1.136)***	3.935 (1.132)***	1.899 (1.150)	
ESD	1.876 (1.314)	1.842 (1.275)	0.925 (1.233)	
NeuroClinic	3.149 (2.128)	4.709 (2.071)**	3.437 (1.996)*	
PatientViews	−1.408 (1.904)	−2.614 (1.897)	−2.639 (1.817)	
Report	0.909 (1.174)	1.088 (1.139)	1.115 (1.092)	
Stroke admissions		−0.000 (0.000)	−0.000 (0.000)	−0.000 (0.000)
Stroke mortality		0.141 (0.129)	0.200 (0.124)	0.230 (0.116)**
No qualifications		0.086 (0.332)	−0.355 (0.336)	−0.177 (0.302)
Median wage		0.084 (0.024)***	0.036 (0.025)	0.057 (0.023)**
Neurosurgeons per region		0.019 (0.026)	0.047 (0.026)*	0.021 (0.023)
Year dummy			6.574 (1.250)***	4.831 (1.178)***
Organisation score				0.331 (0.050)***
AIC	2222.923	2231.788	2205.433	2199.928
BIC	2295.973	2322.657	2299.844	2265.798
Num. obs.	303	303	303	303

*** $p<0.001$, ** $p<0.01$, * $p<0.05$, $^{\cdot }\,p<0.1$. Table 6 shows linear mixed effects results corresponding to the four tree models. The dependent variable is the quality score and explanatory variables are lagged 1 year. Column 1 includes hospital characteristics, human capital factors, teaching and foundation trust status, and primary variables reflecting the quality of the organisation of the process of stroke care. Column 2 adds a set of external variables and column 3 adds the year dummy. Column 4 substitutes the composite organisational measure for the primary organisation features of the process of stroke care

**Table 7:** Correlated random effects results

	Models			
	(1)	(2)	(3)	(4)
(Intercept)	42.975 (5.950)***	6.059 (30.758)	11.222 (30.751)	−19.008 (26.751)
Beds	0.017 (0.017)	0.009 (0.015)	0.008 (0.015)	−0.000 (0.014)
Day case theatres	−0.439 (1.207)	−0.102 (1.047)	−0.230 (1.037)	−0.647 (1.005)
Nurse-to-bed ratio	4.794 (3.921)	0.669 (3.601)	0.552 (3.562)	−1.196 (3.400)
Neurology	−0.648 (0.736)	−1.001 (0.680)	−0.913 (0.674)	−0.994 (0.655)
Neurophysiology	1.669 (3.573)	−0.294 (3.128)	−0.367 (3.094)	−0.085 (3.017)
Neurosurgery	0.794 (1.129)	0.207 (1.041)	0.189 (1.029)	0.053 (1.002)
Teaching	1.761 (1.556)	1.032 (1.538)	1.467 (1.547)	1.110 (1.342)
Foundation trust	2.614 (1.238)**	1.600 (1.257)	1.716 (1.252)	1.675 (1.122)
ASU	1.599 (1.957)	−0.140 (1.862)	−0.198 (1.849)	
RSU	1.107 (1.772)	0.809 (1.708)	0.664 (1.699)	
CSU	3.716 (2.197)*	0.143 (2.123)	0.026 (2.111)	
SUTC	2.389 (1.026)**	2.559 (0.963)***	2.580 (0.956)***	
Specialist	5.394 (1.161)***	2.575 (1.172)**	2.308 (1.170)*	
ESD	2.000 (1.323)	1.216 (1.244)	1.152 (1.235)	
NeuroClinic	3.364 (2.155)	4.069 (2.020)**	3.302 (2.035)	
PatientViews	−1.099 (1.920)	−2.051 (1.851)	−2.378 (1.844)	
Report	0.683 (1.187)	0.674 (1.121)	0.839 (1.116)	
Beds (mean)	−0.021 (0.017)	−0.011 (0.015)	−0.010 (0.015)	−0.003 (0.014)
Day case theatres (mean)	0.349 (1.241)	−0.112 (1.085)	0.072 (1.077)	0.409 (1.033)
Nurse-to-bed ratio (mean)	1.431 (4.314)	3.191 (4.091)	3.294 (4.054)	3.770 (3.824)
Neurology (mean)	0.825 (0.743)	1.099 (0.691)	1.004 (0.684)	1.035 (0.663)
Neurophysiology (mean)	−1.424 (3.634)	0.653 (3.197)	0.785 (3.163)	0.203 (3.073)
Neurosurgery (mean)	−0.881 (1.139)	−0.255 (1.049)	−0.238 (1.038)	−0.094 (1.010)
Stroke admissions		0.030 (0.016)*	0.022 (0.016)	0.015 (0.015)
Stroke mortality		−1.470 (0.958)	−1.209 (0.955)	−0.855 (0.918)
No qualifications		−0.288 (0.366)	−0.322 (0.365)	−0.029 (0.320)
Median wage		0.067 (0.105)	−0.225 (0.169)	−0.133 (0.158)
Neurosurgeons per region		−0.038 (0.201)	−0.224 (0.216)	−0.197 (0.208)
Stroke admissions (mean)		−0.030 (0.016)*	−0.022 (0.016)	−0.015 (0.015)
Stroke mortality (mean)		1.715 (0.974)*	1.416 (0.973)	1.064 (0.933)
Median wage (mean)		−0.025 (0.109)	0.256 (0.168)	0.194 (0.157)
Neurosurgeons per region (mean)		0.077 (0.202)	0.268 (0.218)	0.206 (0.209)
Year dummy			11.414 (5.220)**	10.570 (4.746)**
Organisation score				0.029 (0.083)
Organisation score (mean)				0.449 (0.104)***
AIC	2222.916	2226.488	2218.599	2202.447
BIC	2317.328	2352.433	2348.008	2307.439
Num. obs.	303	303	303	303

*** $p<0.001$, ** $p<0.01$, * $p<0.05$, $^{\cdot }\,p<0.1$. Table 7 shows correlated random effects results for the four models. Each time-varying explanatory variable appears once in its original form and once as a within-group (hospital) average. The coefficients of the variables reflect fixed effects estimates. We control for both hospital and time fixed effects in column 3 and column 4 by adding the year dummy

**Table 8:** Fixed effects model (time)

	Models		
	(1)	(2)	(3)
Beds	−0.004 (0.002)**	−0.002 (0.002)	−0.002 (0.002)
Day case theatres	−0.045 (0.235)	−0.189 (0.237)	−0.246 (0.221)
Nurse-to-bed ratio	4.267 (1.446)***	2.944 (1.567)*	2.201 (1.472)
Neurology	0.189 (0.096)**	0.072 (0.097)	0.037 (0.091)
Neurophysiology	0.302 (0.577)	0.359 (0.570)	0.267 (0.539)
Neurosurgery	−0.069 (0.130)	−0.020 (0.128)	−0.031 (0.119)
Teaching	2.193 (1.381)	1.710 (1.359)	1.181 (1.242)
Foundation trust	1.405 (1.138)	1.772 (1.160)	2.042 (1.074)*
ASU	−0.755 (1.857)	−0.245 (1.836)	
RSU	1.605 (1.628)	0.632 (1.627)	
CSU	0.958 (2.073)	0.668 (2.033)	
SUTC	2.902 (0.952)***	3.261 (0.936)***	
Specialist	2.381 (1.158)**	2.047 (1.147)*	
ESD	1.248 (1.236)	1.248 (1.225)	
NeuroClinic	2.893 (1.998)	4.110 (1.985)**	
PatientViews	−1.171 (1.779)	−2.386 (1.808)	
Report	1.338 (1.100)	1.197 (1.084)	
Stroke admissions		−0.000 (0.000)	−0.000 (0.000)
Stroke mortality		0.216 (0.125)*	0.238 (0.115)**
No qualifications		−0.347 (0.306)	−0.154 (0.284)
Median wage		0.036 (0.023)	0.059 (0.021)***
Neurosurgeon per region		0.045 (0.023)*	0.018 (0.021)
Organisation score			0.359 (0.049)***
R$^2$	0.191	0.244	0.297
Adj. R$^2$	0.179	0.225	0.281
Num. obs.	303	303	303

*** $p<0.001$, ** $p<0.01$, * $p<0.05$, $^{\cdot }\,p<0.1$. Column 1 includes the internal drivers of clinical quality of the process of stroke care. Column 2 adds potential external drivers. Column 3 replaces the individual organisational variables with the composite measure of organisational quality. All the three models control for time fixed effects

**Table 9:** Correlated random effects results: rescaled clinical and organisational quality scores

	Models			
	(1)	(2)	(3)	(4)
(Intercept)	−9.850 (11.11)	−53.01 (20.19)***	−50.61 (27.51)*	−55.67 (26.95)**
Beds	−0.003 (0.002)*	−0.002 (0.002)	−0.001 (0.002)	−0.002 (0.002)
Day case theatres	−0.125 (0.204)	−0.228 (0.207)	−0.352 (0.266)	−0.228 (0.207)
Nurse-to-bed ratio	2.358 (1.475)	1.853 (1.408)	1.116 (1.538)	1.864 (1.415)
Neurology	0.070 (0.079)	0.035 (0.079)	−0.023 (0.137)	0.037 (0.079)
Neurophysiology	0.320 (0.453)	0.297 (0.419)	−0.067 (0.617)	0.294 (0.420)
Neurosurgery	−0.044 (0.116)	−0.024 (0.100)	0.029 (0.112)	−0.026 (0.101)
Teaching	1.414 (1.330)	1.163 (1.300)	1.514 (1.518)	1.186 (1.322)
Foundation trust	1.248 (0.994)	1.849 (1.015)*	1.811 (1.115)	1.841 (1.019)*
Stroke admissions	−0.000 (0.000)	−0.000 (0.000)	−0.000 (0.000)	−0.000 (0.000)
Stroke mortality	0.084 (0.105)	0.228 (0.122)*	0.204 (0.138)	0.237 (0.136)*
No qualifications	−0.422 (0.321)	−0.182 (0.301)	−0.173 (0.309)	−0.189 (0.306)
Neurosurgeons per region	0.060 (0.015)***	0.022 (0.020)	0.028 (0.038)	0.028 (0.038)
Median wage		0.056 (0.020)***	0.060 (0.032)*	0.060 (0.031)*
Organisation score	0.297 (0.048)***	0.325 (0.049)***	0.313 (0.053)***	0.326 (0.049)***
London				−1.042 (5.961)
Year dummy	−0.502 (0.965)	−1.668 (1.020)	−1.540 (1.323)	−1.782 (1.246)
*R* $^2$	0.230	0.250	0.143	0.250
Adj.* R* $^2$	0.218	0.237	0.134	0.236
Num. obs.	303	303	265	303

*** $p<0.01$, ** $p<0.05$, * $p<0.1$. The dependent variable in all the models is the change in the quality of the process of stroke care obtained by taking out the within-hospital mean of the variable from the variable. Similarly, the table replaces the composite organisational score with the change in the variable obtained by taking out the within-hospital mean of the variable. Column 3 excludes data relating to London hospitals. Column 4 includes the London dummy, using the full data

**Table 10:** Fixed effects models: organisation score

	Models			
	(1)	(2)	(3)	(4)
Beds	0.017 (0.012)	0.018 (0.012)	0.018 (0.012)	0.016 (0.012)
Day case theatres	1.112 (0.790)	0.930 (0.795)	0.963 (0.798)	1.004 (0.788)
Nurse-to-bed ratio	5.238 (2.987)*	6.486 (3.079)**	6.301 (3.106)**	5.519 (3.086)*
Neurology	−0.260 (0.576)	−0.084 (0.585)	−0.112 (0.588)	−0.202 (0.582)
Neurophysiology	1.554 (2.918)	1.857 (2.912)	1.851 (2.924)	1.209 (2.901)
Neurosurgery	−0.558 (0.904)	−0.423 (0.904)	−0.483 (0.904)	−0.099 (0.909)
Teaching	0.540 (10.373)	0.490 (10.327)	0.466 (10.357)	0.865 (10.227)
Foundation trust	−2.747 (1.847)	−1.354 (2.044)	−1.718 (2.026)	−0.764 (2.045)
Median wage			−0.034 (0.028)	0.358 (0.176)**
*R* $^2$	0.055	0.051	0.064	0.076
Adj.* R* $^2$	0.026	0.024	0.029	0.035
Num. obs.	342	342	342	342

*** $p<0.001$, ** $p<0.01$, * $p<0.05$, $^{\cdot }\,p<0.1$. The dependent variable is the composite organisational measure, all explanatory variables are lagged for 1 year, and the coefficients are all fixed effects estimates. Column 1 controls for hospital fixed effects while column 2 controls for hospital and time fixed effects. Columns 3 and 4 add weekly median wage. The columns respectively control for individual fixed effects and both individual and time fixed effects. Column 5 adds the lag of the dependent variable and controls for individual and time fixed effects

## References

[1] Aragon, M.J.A., Castelli, A., Gaughan, J.: Hospital trusts productivity in the english nhs: uncovering possible drivers of productivity variations. CHE University of York Research Paper 117 (2015) 10.1371/journal.pone.0182253 PMC5540600 28767731

[2] Athey, S., Stern, S.: An empirical framework for testing theories about complimentarity in organizational design. NBER Working Paper, National Bureau of Economic Research (1998)

[3] Ayanian JZ, Weissman JS (2002). Teaching hospitals and quality of care: a review of the literature. Milbank Q..

[4] Bijlsma MJ, Koning PW, Shestalova V (2013). The effect of competition on process and outcome quality of hospital care in the Netherlands. De Econ..

[5] Birkmeyer JD, Dimick JB (2009). Understanding and reducing variation in surgical mortality. Annu. Rev. Med..

[6] Bloom, N., Lemos, R., Sadun, R., Scur, D., Reenen, J.V.: The new empirical economics of management. CEP Occasional Papers 41, Centre for Economic Performance, LSE (2014)

[7] Bloom N, Propper C, Seiler S, Van Reenen J (2015). The impact of competition on management quality: evidence from public hospitals. Rev. Econ. Stud..

[8] Bloom, N., Sadun, R., Van Reenen, J.: Does management matter in healthcare? Stanford Mimeo (2014)

[9] Bloom N, Van Reenen J (2007). Measuring and explaining management practices across firms and countries. Q. J. Econ..

[10] Bradley EH, Curry LA, Spatz ES, Herrin J, Cherlin EJ, Curtis JP, Thompson JW, Ting HH, Wang Y, Krumholz HM (2012). Hospital strategies for reducing risk-standardized mortality rates in acute myocardial infarction. Ann. Intern. Med..

[11] Bradley EH, Herrin J, Curry L, Cherlin EJ, Wang Y, Webster TR, Drye EE, Normand S-LT, Krumholz HM (2010). Variation in hospital mortality rates for patients with acute myocardial infarction. Am. J. Cardiol..

[12] Bray BD, Ayis S, Campbell J, Hoffman A, Roughton M, Tyrrell PJ, Wolfe CD, Rudd AG (2013). Associations between the organisation of stroke services, process of care, and mortality in England: prospective cohort study. BMJ.

[13] Breiman L, Friedman J, Stone CJ, Olshen RA (1984). Classification and Regression Trees.

[14] Brynjolfsson E, Milgrom P, Gibbons R, Roberts J (2013). Complementarity in organization. The Handbook of Organizational Economics.

[15] Burnham KP, Anderson DR (2002). Model Selection and Multimodel Inference: A Practical Information-theoretic Approach.

[16] Candelise, L., Gattinoni, M., Bersano, A., Micieli, G., Sterzi, R., Morabito, A., the PROSIT Study Group, et al.: Lancet stroke-unit care for acute stroke patients: an observational follow-up study. **369**(9558), 299–305 (2007) 10.1016/S0140-6736(07)60152-4 17258670

[17] Caplan L (2003). Stroke is best managed by neurologists. Stroke.

[18] Chandra A, Finkelstein A, Sacarny A, Syverson C (2016). Productivity dispersion in medicine and manufacturing. Am. Econ. Rev..

[19] Chen LM, Nallamothu BK, Krumholz HM, Spertus JA, Tang F, Chan PS (2013). Association between a hospital’s quality performance for in-hospital cardiac arrest and common medical conditions. Circ. Cardiovasc. Qual. Outcomes.

[20] Cho S-H, Yun S-C (2009). Bed-to-nurse ratios, provision of basic nursing care, and in-hospital and 30-day mortality among acute stroke patients admitted to an intensive care unit: cross-sectional analysis of survey and administrative data. Int. J. Nurs. Stud..

[21] Cooper RA (2009). Regional variation and the affluence-poverty nexus. JAMA.

[22] Crombie I, Davies H (1998). Beyond health outcomes: the advantages of measuring process. J. Eval. Clin. Pract..

[23] Farrar S, Yi D, Sutton M, Chalkley M, Sussex J, Scott A (2013). Has payment by results affected the way that English Hospitals provide care? Difference–in–differences analysis. BMJ.

[24] Fine JM, Fine MJ, Galusha D, Petrillo M, Meehan TP (2002). Patient and hospital characteristics associated with recommended processes of care for elderly patients hospitalized with pneumonia: results from the medicare quality indicator system pneumonia module. Arch. Intern. Med..

[25] Flood AB (1994). The impact of organizational and managerial factors on the quality of care in health care organizations. Med. Care Res. Rev..

[26] Friedman, M.: Essays in positive economics. University of Chicago Press (1953)

[27] Fu W, Simonoff JS (2015). Unbiased regression trees for longitudinal and clustered data. Comput. Stat. Data Anal..

[28] Garicano L, Rayo L (2016). Why organizations fail: models and cases. J. Econ. Lit..

[29] Gaynor M, Laudicella M, Propper C (2012). Can governments do it better? Merger mania and hospital outcomes in the english nhs. J. Health Econ..

[30] Gaynor M, Seider H, Vogt WB (2005). The volume-outcome effect, scale economies, and learning-by-doing. Am. Econ. Rev..

[31] Ghaferi AA, Birkmeyer JD, Dimick JB (2009). Variation in hospital mortality associated with inpatient surgery. New Engl. J. Med..

[32] Gobillon L, Milcent C (2013). Spatial disparities in hospital performance. J. Econ. Geogr..

[33] Grimaud O, Béjot Y, Heritage Z, Vallée J, Durier J, Cadot E, Giroud M, Chauvin P (2011). Incidence of stroke and socioeconomic neighborhood characteristics an ecological analysis of dijon stroke registry. Stroke.

[34] Hentschker, C., Mennicken, R.: The volume–outcome relationship and minimum volume standards–empirical evidence for Germany. Health Econ. (2014) 10.1002/hec.3051 24700615

[35] Hoeks, S., Scholte op Reimer, W., Lingsma, H., van Gestel, Y., van Urk, H., Bax, J., Simoons, M., Poldermans, D.: Process of care partly explains the variation in mortality between hospitals after peripheral vascular surgery. Eur. J. Vasc. Endovasc. Surg. **40**(2), 147–154 (2010) 10.1016/j.ejvs.2010.04.005 20547077

[36] Hothorn T, Hornik K, Zeileis A (2006). Unbiased recursive partitioning: a conditional inference framework. J. Comput. Graph. Stat..

[37] Kapral MK, Fang J, Chan C, Alter DA, Bronskill SE, Hill MD, Manuel DG, Tu JV, Anderson GM (2012). Neighborhood income and stroke care and outcomes. Neurology.

[38] Katz ML (2013). Provider competition and healthcare quality: more bang for the buck?. Int. J. Indus. Organ..

[39] Ketcham JD, Baker LC, MacIsaac D (2007). Physician practice size and variations in treatments and outcomes: evidence from medicare patients with ami. Health Affairs.

[40] Kolstad JT, Kowalski AE (2012). The impact of health care reform on hospital and preventive care: evidence from massachusetts. J. Public Econ..

[41] Kuhn M, Johnson K (2013). Applied Predictive Modeling.

[42] Kupersmith J (2005). Quality of care in teaching hospitals: a literature review. Acad. Med..

[43] Langhorne P, Taylor G, Murray G, Dennis M, Anderson C, Bautz-Holter E, Dey P, Indredavik B, Mayo N, Power M (2005). Early supported discharge services for stroke patients: a meta-analysis of individual patients’ data. Lancet.

[44] Lindenauer PK, Remus D, Roman S, Rothberg MB, Benjamin EM, Ma A, Bratzler DW (2007). Public reporting and pay for performance in hospital quality improvement. New Engl. J. Med..

[45] Manheim LM, Feinglass J, Shortell SM, Hughes EF (1992). Regional variation in medicare hospital mortality. Inquiry.

[46] May K (1947). Technological change and aggregation. Econometrica.

[47] McClellan, M.B., Staiger, D.O.: Comparing hospital quality at for-profit and not-for-profit hospitals. In: The changing hospital industry: comparing for-profit and Not-for-profit institutions, pp. 93–112. University of Chicago Press (2000)

[48] McConnell KJ, Chang AM, Maddox TM, Wholey DR, Lindrooth RC (2014). An exploration of management practices in hospitals. Healthcare.

[49] McConnell KJ, Lindrooth RC, Wholey DR, Maddox TM, Bloom N (2013). Management practices and the quality of care in cardiac units. JAMA Intern. Med..

[50] McNaughton H, McPherson K, Taylor W, Weatherall M (2003). Relationship between process and outcome in stroke care. Stroke.

[51] Menachemi N, Chukmaitov A, Saunders C, Brooks RG (2008). Hospital quality of care: does information technology matter? The relationship between information technology adoption and quality of care. Health Care Manag. Rev..

[52] Milgrom P, Roberts J (1995). Complementarities and fit strategy, structure, and organizational change in manufacturing. J. Account. Econ..

[53] Mitchell JB, Ballard DJ, Whisnant JP, Ammering CJ, Samsa GP, Matchar DB (1996). What role do neurologists play in determining the costs and outcomes of stroke patients?. Stroke.

[54] Mohammed MA, Mant J, Bentham L, Raftery J (2005). Comparing processes of stroke care in high-and low-mortality hospitals in the West Midlands, UK. Int. J. Qual. Health Care.

[55] Mooney H (2010). Quality of stroke care varies widely across England. BMJ.

[56] Needleman J, Buerhaus P, Mattke S, Stewart M, Zelevinsky K (2002). Nurse-staffing levels and the quality of care in hospitals. New Engl. J. Med..

[57] Needleman J, Buerhaus P, Pankratz VS, Leibson CL, Stevens SR, Harris M (2011). Nurse staffing and inpatient hospital mortality. New Engl. J. Med..

[58] O’Brien SM, DeLong ER, Peterson ED (2008). Impact of case volume on hospital performance assessment. Arch. Intern. Med..

[59] Ogbu UC, Slobbe LC, Arah OA, de Bruin A, Stronks K, Westert GP (2010). Hospital stroke volume and case-fatality revisited. Med. Care.

[60] Park S, Lee J, Ikai H, Otsubo T, Imanaka Y (2013). Decentralization and centralization of healthcare resources: investigating the associations of hospital competition and number of cardiologists per hospital with mortality and resource utilization in japan. Health Policy.

[61] Propper C (2012). Competition, incentives and the english nhs. Health Econ..

[62] Propper C, Burgess S, Green K (2004). Does competition between hospitals improve the quality of care? Hospital death rates and the nhs internal market. J. Public Econ..

[63] Quinlan JR (2014). C4.5: Programs for Machine Learning.

[64] Rafferty AM, Clarke SP, Coles J, Ball J, James P, McKee M, Aiken LH (2007). Outcomes of variation in hospital nurse staffing in english hospitals: cross-sectional analysis of survey data and discharge records. Int. J. Nurs. Stud..

[65] Reeves MJ, Gargano J, Maier KS, Broderick JP, Frankel M, LaBresh KA, Moomaw CJ, Schwamm L (2010). Patient-level and hospital-level determinants of the quality of acute stroke care a multilevel modeling approach. Stroke.

[66] Ringel SP (1996). The neurologist’s role in stroke management. Stroke.

[67] Ringelstein EB, Chamorro A, Kaste M, Langhorne P, Leys D, Lyrer P, Thijs V, Thomassen L, Toni D (2013). European stroke organisation recommendations to establish a stroke unit and stroke center. Stroke.

[68] Roland M, Rosen R (2011). English nhs embarks on controversial and risky market-style reforms in health care. New Engl. J. Med..

[69] Rudd AG, Irwin P, Rutledge Z, Lowe D, Wade D, Pearson M (2001). Regional variations in stroke care in England, wales and Northern Ireland: results from the national sentinel audit of stroke. Clin. Rehabil..

[70] Saposnik G, Baibergenova A, O’Donnell M, Hill M, Kapral M, Hachinski V (2007). Hospital volume and stroke outcome does it matter?. Neurology.

[71] Schootman M, Lian M, Pruitt SL, Deshpande AD, Hendren S, Mutch M, Jeffe DB, Davidson N (2014). Hospital and geographic variability in thirty-day all-cause mortality following colorectal cancer surgery. Health Serv. Res..

[72] Sela RJ, Simonoff JS (2012). Re-em trees: a data mining approach for longitudinal and clustered data. Mach. Learn..

[73] Svendsen ML, Ehlers LH, Frydenberg M, Ingeman A, Johnsen SP (2011). Quality of care and patient outcome in stroke units: is medical specialty of importance?. Med. Care.

[74] Svendsen ML, Ehlers LH, Ingeman A, Johnsen SP (2012). Higher stroke unit volume associated with improved quality of early stroke care and reduced length of stay. Stroke.

[75] Syverson C (2011). What determines productivity?. J. Econ. Literat..

[76] Ukawa N, Ikai H, Imanaka Y (2014). Trends in hospital performance in acute myocardial infarction care: a retrospective longitudinal study in japan. Int. J. Qual. Health Care.

[77] West E (2001). Management matters: the link between hospital organisation and quality of patient care. Qual. Health Care.

[78] Whisnant JP (1983). The role of the neurologist in the decline of stroke. Ann. Neurol..

[79] Wooldridge J (2012). Introductory Econometrics: A Modern Approach.

[80] Xian Y, Holloway RG, Chan PS, Noyes K, Shah MN, Ting HH, Chappel AR, Peterson ED, Friedman B (2011). Association between stroke center hospitalization for acute ischemic stroke and mortality. JAMA.

